# Early Enzyme Replacement Therapy Improves Hearing and Immune Defects in Adenosine Deaminase Deficient-Mice

**DOI:** 10.3389/fimmu.2019.00416

**Published:** 2019-03-13

**Authors:** Xiaobai Xu, Jaina Negandhi, Weixian Min, Michael Tsui, Martin Post, Robert V. Harrison, Eyal Grunebaum

**Affiliations:** ^1^Institute of Medical Science, University of Toronto, Toronto, ON, Canada; ^2^Developmental and Stem Cell Biology Program, Hospital for Sick Children, Toronto, ON, Canada; ^3^Neuroscience and Mental Health Program, Hospital for Sick Children, Toronto, ON, Canada; ^4^Translational Medicine Program, Hospital for Sick Children, Toronto, ON, Canada; ^5^Department of Laboratory Medicine & Pathology, Hospital for Sick Children, Toronto, ON, Canada; ^6^Department of Otolaryngology, University of Toronto, Toronto, ON, Canada; ^7^Division of Immunology and Allergy, Hospital for Sick Children, Toronto, ON, Canada

**Keywords:** adenosine deaminase deficiency, hearing, enzyme replacement, PEG-ADA, enzyme replacement therapy

## Abstract

**Background:** Inherited defects in adenosine deaminase (ADA) cause severe immune deficiency, which can be corrected by ADA enzyme replacement therapy (ERT). Additionally, ADA-deficient patients suffer from hearing impairment. We hypothesized that ADA-deficient (–/–) mice also exhibit hearing abnormalities and that ERT from an early age will improve the hearing and immune defects in these mice.

**Methods:** Auditory brainstem evoked responses, organ weights, thymocytes numbers, and subpopulations, lymphocytes in peripheral blood as well as T lymphocytes in spleen were analyzed in ADA–/– and ADA-proficient littermate post-partum (pp). The cochlea was visualized by scanning electron microscopy (SEM). The effects of polyethylene glycol conjugated ADA (PEG-ADA) ERT or 40% oxygen initiated at 7 days pp on the hearing and immune abnormalities were assessed.

**Results:** Markedly abnormal hearing thresholds responses were found in ADA–/– mice at low and medium tone frequencies. SEM demonstrated extensive damage to the cochlear hair cells of ADA–/– mice, which were splayed, short or missing, correlating with the hearing deficits. The hearing defects were not reversed when hypoxia in ADA–/– mice was corrected. Progressive immune abnormalities were detected in ADA–/– mice from 4 days pp, initially affecting the thymus followed by peripheral lymphocytes and T cells in the spleen. ERT initiated at 7 days pp significantly improved the hearing of ADA–/– mice as well as the number of thymocytes and T lymphocytes, although not all normalized.

**Conclusions:** ADA deficiency is associated with hearing deficits and damage to cochlear hair cells. Early initiation of ERT improves the hearing and immune abnormalities.

## Introduction

Adenosine deaminase (ADA) is a ubiquitous enzyme important for the degradation and salvage of purine metabolites, including adenosine (Ado) and deoxyadenosine (dAdo). The inherited absence of ADA leads to accumulation of these metabolites that are toxic to rapidly proliferating cells such as lymphocytes (1), resulting in severe combined immunodeficiency (SCID). ADA-deficient SCID (ADA-SCID) patients typically present in the first months of life with increased susceptibility to infections (2). Additionally, patients often suffer from non-infectious complications including skeletal dysplasia (3, 4), renal mesangial sclerosis (5), hepatic failure (6), myeloid abnormalities (7), and pulmonary alveolar proteinosis (8). Diverse neurological abnormalities, such as seizures, developmental delay, and cognitive impairment have frequently been reported among ADA-deficient patients (9–11). Additionally, sensory-neural auditory abnormalities have been identified in ADA-deficient patients (12, 13), however, the hearing defects have not been well-characterized and their pathogenesis is not clear. Infections, autoimmunity, hypoxia, or increased susceptibility of the auditory structures to medications used to treat ADA-SCID patients have been proposed as a potential cause(s) for the hearing deficits. Alternatively, the abnormal hearing in ADA deficiency might be directly caused by the altered Ado metabolism.

ADA deficiency can be treated by different treatment modalities. The immune defects are corrected by hematopoietic stem cells transplants (HSCT) using ADA-proficient allogeneic cells or autologous *ex-vivo* gene-corrected cells (14). Lung abnormalities also improve following HSCT (8). However, the neurological and hearing defects often persist, possibly because of the irreversible damage occurring prior to treatment (12, 15). The ability of purines to cross cells' membranes through concentration-dependent and -independent transporters led to the development of enzyme replacement therapy (ERT) for ADA deficiency (16). ERT is performed by frequent injections of polyethylene glycol coupled to ADA (PEG-ADA), as PEGilation reduces the immunogenicity of the bovine-based ADA and increases the biological half-life of the enzyme (17, 18). ERT has been shown to rapidly reverse the metabolic abnormalities, and improve immune function (19). Moreover, ERT can correct the lung abnormalities (8), liver function (6), and bone dysplasia (4) in ADA-SCID. Yet, the increase in peripheral blood T cells numbers and function is often delayed for several months after initiation of ERT (19). This has raised concerns about the immediate benefits of ERT, particularly when used for short periods as a “bridge” until HSCT. The interest in short-term ERT has become of even greater importance with the increasing number of ADA-deficient infants diagnosed promptly after birth through wide-spread newborn screening (NBS) for SCID programs (20). In addition, while the effects of ERT on the neurological abnormalities associated with ADA deficiency have been explored recently (11), little is known about the benefits for the hearing defects. A recent publication found that 4 of 16 ADA-deficient patients treated with ERT had hearing deficits including auditory brainstem evoked response (ABR) abnormalities (11). However, ERT was not uniformly provided, hindering the ability to assess its effectiveness.

Mice lacking ADA activity (ADA–/–) exhibit many of the metabolic and immune and non-infectious abnormalities observed in ADA-SCID patients including liver disease, skeletal malformations and renal sclerosis (21). Moreover, by 14 days post-partum (pp) ADA–/– mice develop alveolar proteinosis, leading to severe lung disease, hypoxia, and ultimate death by 3 weeks of age (22). Previous studies of the brains of ADA–/– mice at 17 days pp demonstrated marked enlargement of the ventricles, possibly related to hypoxia, with normal myelination and no evidence for neuronal loss (11, 23), however, hearing was not evaluated in these mice.

ADA–/– mice have also been instrumental in assessing HSCT and gene therapy for ADA deficiency (24–26). Early ERT has also been previously described to improve immune function in these mice, twice weekly injections of PEG-ADA at high doses (1–5 units of PEG-ADA per gram body weight) from day 10 pp can normalize the percentages of CD4+CD8+ cells in the thymus of 7–8 weeks old ADA–/– mice and prevent the respiratory failure (24). In contrast, PEG-ADA initiated at 10 days pp doesn't improve the behavioral deficits, such as exploration and anxiety-like activity in ADA–/– mice, despite a marked reduction in the accumulation of Ado in the mice brain (11), raising concerns on the benefits of ERT for the neurological defects.

Because of the similarities of ADA deficiency in human and mice, we hypothesized that ADA–/– mice might also develop hearing abnormalities, which would enable a better understanding of the mechanisms involved in such process. Moreover, we reasoned that initiation of ERT shortly after birth could prevent the progressive auditory and immune abnormalities in ADA–/– mice, thereby providing additional support for the early use of ERT in ADA-SCID.

## Methods

ADA–/– (FVB, 129-Adatm1Mw-TgN[PLADA]) mice and ADA+/− littermate control mice were maintained in a pathogen-free environment, as previously described (22). ADA activity was assessed in fresh blood samples obtained from the tails of 7 days pp mice by conversion of [14C] Ado (Moravek biochemical, Brea, CA, USA) to inosine, followed by TLC separation, as previously reported (22). All animal procedures were approved by the Institute's Animal Care Committee and performed in accordance with the Canadian Council for Animal Care guidelines.

Mice received ERT with PEG-ADA (ADAGEN®; ENZON Pharmaceuticals Inc., Piscataway, NJ, USA, Sigma-Tau Pharmaceuticals, Inc.), or the equivalent volume of PBS (“untreated”) using 2 regimens. In the “early” ERT regimen, mice received 1 unit/gram body weight of PEG-ADA by intraperitoneal injections twice a week from 7 days pp and continued until 21 days pp. In the “late” regimen, PEG-ADA was initiated at 10 days pp and administered once a week, as recently described (11). One unit of PEG-ADA was defined as the amount of enzyme necessary to convert 1 μM of Ado to inosine per minute at 25°C.

Some ADA–/– and ADA+/– mice were transferred at 7 days pp to Oxycycler Exposure Chambers (Biospherix Ltd, Lacona, NY, USA) equipped with ProOx 110 controller (Biospherix), which provided 40% oxygen, as previously described (27). Oxygen concentrations were monitored continuously and maintained in the chamber at all times. The chambers were regulated with a 12 h light-dark cycle. Food and water were supplied *ad libitum*. Litter sizes were kept at no more than 6 pups in both the hyperoxia and room air groups. Oxygen saturation in the arterial blood of mice was analyzed using the ABL800 FLEX blood gas analyzer (Radiometer, Copenhagen, Denmark).

ABR recording was performed as described previously (28). Briefly, mice were anesthetized with i.p. injection of ketamine (100 mg/kg) and xylazine (10 mg/kg). ABR was recorded with needle skin electrodes in a vertex/mastoid configuration. Signals were amplified (x1,000), filtered (0.1–3 kHz) and averaged (512 sweeps; Intelligent Hearing Systems Miami, Fl. USA). ABR in response to short gated tones at 8, 16, and 32 kHz was measured in each ear. Stimuli were presented using a high-frequency transducer connected in a closed system to the external ear canal. Stimuli were presented in the range of 90–0 dB sound pressure levels in 5 or 10 dB intensity steps. ABR thresholds were determined by visual inspection of the ABR waveforms.

Scanning electron microscopy (SEM) of the cochlea was carried out with the following steps: inner ear dissection, tissue fixation in 2.5% glutaraldehyde, specimen dehydration, cochlear micro-dissection to reveal the sensory epithelium, critical point drying, gold sputter coating, and scanning using the Hitachi S3400 electron microscope. The morphology of the sensory epithelium of the organ of Corti, particularly the integrity of hair cells in basal, middle, and apical cochlear turns was evaluated.

Lymphocyte numbers in the blood were determined using the Hemavet 950FS. The weight of the thymus and spleen were recorded. Lymphocyte subpopulations isolated from the thymus and spleen of mice were characterized by the expression of CD3, CD4, and CD8 among viable cells, as previously described (29).

Values are expressed as mean ± SD. The statistical significance of differences between 2 groups was determined by using the 2-tailed Student *t*-test by using Microsoft Excel software (Microsoft, Redmond, Wash). Differences among 3 or more groups were determined by using ANOVA with Tukey *post-hoc* comparison adjustment for multiple comparisons using Stata software (StataCorp LP, College Station, Tex). A *p* < 0.05 was considered statistically significant.

## Results

**Figure 1:**
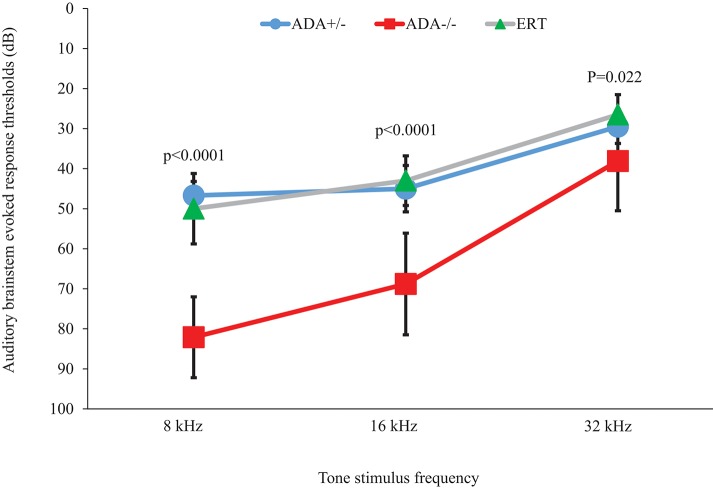
Audiograms of ADA+/– mice, ADA–/– mice, and ADA–/– mice following ERT. Auditory brainstem evoked response thresholds at 8, 16, and 32 kHz tone stimulus in 17 days post-partum ADA–/– mice (red squares; *n* = 7), ADA+/– littermate control mice (blue circles; *n* = 8), and ADA–/– mice receiving ERT (green triangles; *n* = 10). Results are the mean and standard deviation of 5 independent experiments.

Hearing onset in mice occurs at 11–13 days pp (30) and ADA–/– mice suffer respiratory failure and death by 19–21 days pp, therefore ABR testing was performed at 17 days pp. As shown in Figure 1, ADA–/– mice demonstrated significant (*p* < 0.0001) hearing loss (increase in auditory threshold) compared to ADA+/– littermate control mice at 8 and 16 kHz, while at 32 kHz the differences were significant yet not as pronounced. To further establish a direct role of abnormal purine metabolism in the hearing abnormalities, ADA–/– mice were treated with ERT. In preliminary studies the optimal PEG-ADA administration schedule was determined by measuring ADA activity in the blood of 15 days pp ADA–/– mice (*n* = 5 in each group) treated with “early” or “late” ERT regimens. ADA activity was significantly higher (*p* < 0.0001) with the “early” regimen (156.7 ± 12.4% of the activity in healthy littermate mice) compared to mice treated in accordance to the “late” regimen (34.5 ± 4.1% of activity in healthy littermate mice). Therefore, in subsequent studies, the “early” ERT regimen was utilized. Early ERT improvements of ABR in ADA–/– mice were particularly evident at 8 kHz (Figure 1), where the threshold decreased significantly from 82.2 ± 10.1 to 50.3 ± 8.8 dB (*p* < 0.0001) and at 16 kHz where the threshold decreased from 68.8 ± 12.7 to 43.5 ± 6.2 dB (*p* < 0.0001). At 32 kHz there was also a significant improvement in hearing threshold (*p* = 0.022), although not as prominent as at lower frequencies.

To exclude the possibility that the hearing abnormalities observed at 17 days pp ADA–/– mice were due to delayed development of hearing, mice were further treated until 21 days pp with ERT, thereby extending their survival and enabling ABR testing at 31–35 days pp. Hearing threshold of these ADA–/– mice (*n* = 5) at 31–35 days old were still increased at both 8 and 16 kHz (66.3 ± 13.6 and 58.5 ± 18.5 dB, respectively) compared to ADA+/– littermates controls, indicating that the hearing abnormalities were not due to a developmental delay.

**Figure 2:**
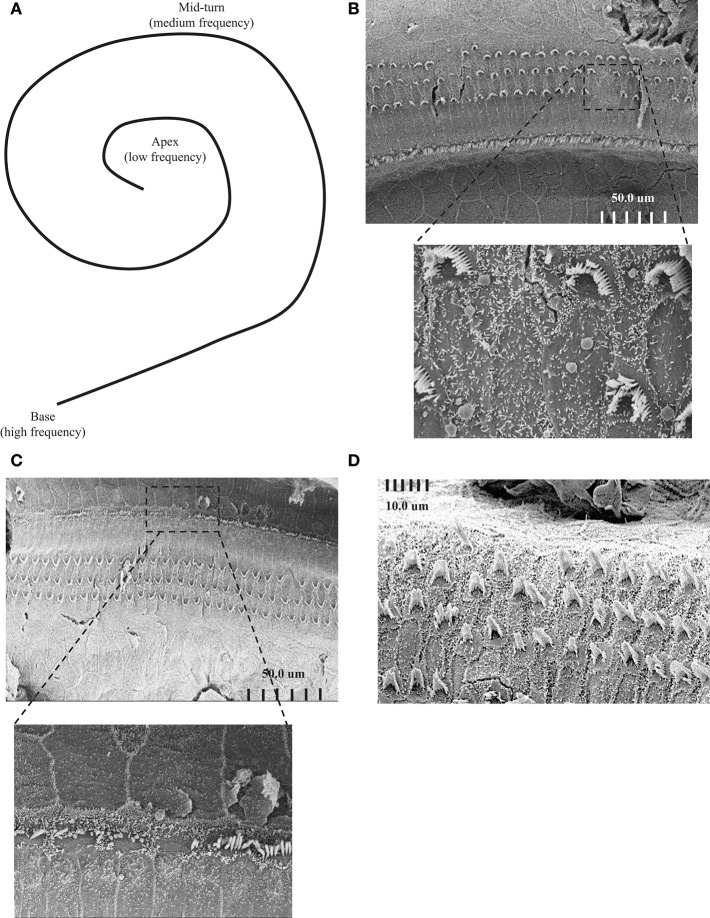
Scanning electron microscopy (SEM) images of ADA–/– and ADA+/– mice. **(A)** Schematic illustration of the cochlea including the apex, the mid-turn and the base areas, corresponding to low, medium, and high frequency hearing. **(B)** Representative SEM of the sensory epithelium of the cochlear apex of 17 days post-partum (pp) ADA–/– mice demonstrating loss of inner and outer hair cells and much shorter stereocilia length (Magnifications X950). The bottom image is a higher magnification (X6500) of the upper micrograph boxed region. **(C)** Representative SEM of the sensory epithelium of the cochlear mid-turn region of 17 days post-partum (pp) ADA–/– mice (Magnifications X850). The bottom image is a higher magnification (X4200) of the upper micrograph boxed region. **(D)** Representative SEM of the sensory epithelium of the cochlear apex of 17 days post-partum (pp) ADA+/– littermate control mice. Magnification X3000.

**Figure 3:**
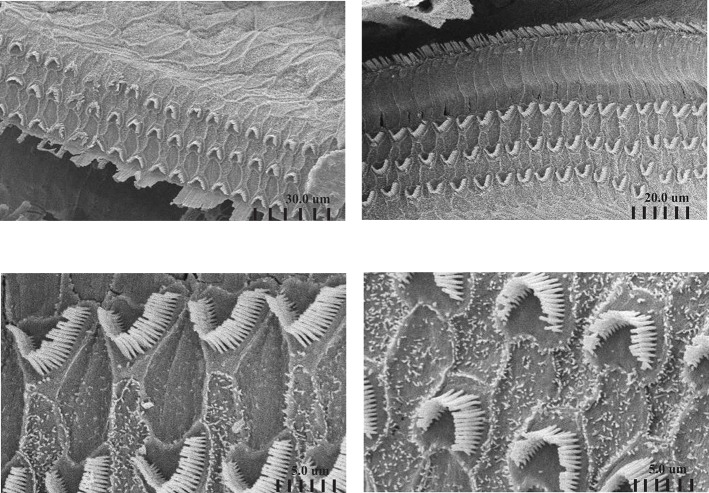
Scanning electron microscopy of the cochlea of ADA–/– mice following ERT. Representative SEM of the sensory epithelium of the cochlear apex of 17 days post-partum (pp) ADA–/– mice treated with early ERT demonstrating normal appearing hair cells (top panels) and normal length stereocilia (bottom panels). Magnifications: top left X 1800, top right X 2000, bottom left X8500, bottom right X9000.

To further understand the effect of ADA deficiency on the inner ear, the structure of the cochlea was assessed using SEM. Attention was focused on the integrity of hair cells at the cochlear apex and mid-turn, activated by low and medium frequency tones, respectively, in comparison to the basal cochlear region where high-frequency sound stimuli are transduced, as illustrated in Figure 2A. SEM revealed damage to the sensory epithelium at the apical (Figure 2B) and mid-turn (Figure 2C) of the cochlea in 17 days pp ADA–/– mice, regions corresponding to the ABR threshold losses at 8 and 16 kHz, respectively. The changes in ADA–/– mice included splaying and loss of inner and outer hair cells as well as much shorter stereocilia length, in comparison with ADA+/– control mice (Figure 2D). Consistent with the less severe hearing abnormalities at 32 kHz, hair cells in the basal regions of the cochlea in 17 days pp ADA–/– mice were not markedly affected (data not shown). Moreover, early ERT reversed the SEM abnormalities observed in the cochlea of ADA–/– mice. As illustrated in Figure 3, the mid-turn region of the cochlea in ADA–/– mice following PEG-ADA treatment had normal-appearing hair cells morphology with no hair cell loss and with normal length stereocilia.

ADA–/– mice suffer from progressive lung abnormalities and hypoxemia at 18 days pp (22). To determine the potential role of hypoxia in the hearing loss in ADA deficiency, ADA–/– mice were housed from 7 days pp in chambers providing 40% oxygen. Oxygen treatment significantly (*p* = 0.011) improved the oxygen saturation in the blood of ADA–/– mice from 84.0 ± 3.8% (*n* = 4) to 90.8 ± 4.7% (*n* = 7), which was not significantly different than the 92.3 ± 3.9% saturation in littermate control mice (*n* = 5). However, ABR impairment of ADA–/– mice persisted at 8 and 16 kHz (thresholds of 82.2 ± 11.8 and 73.3 ± 15.2 dB, respectively) and were not significantly different than the thresholds abnormalities in ADA–/– mice housed without oxygen supplementation. Hence, these results suggest that systemic hypoxia is not the major cause for the hearing defects in ADA–/– mice.

**Figure 4:**
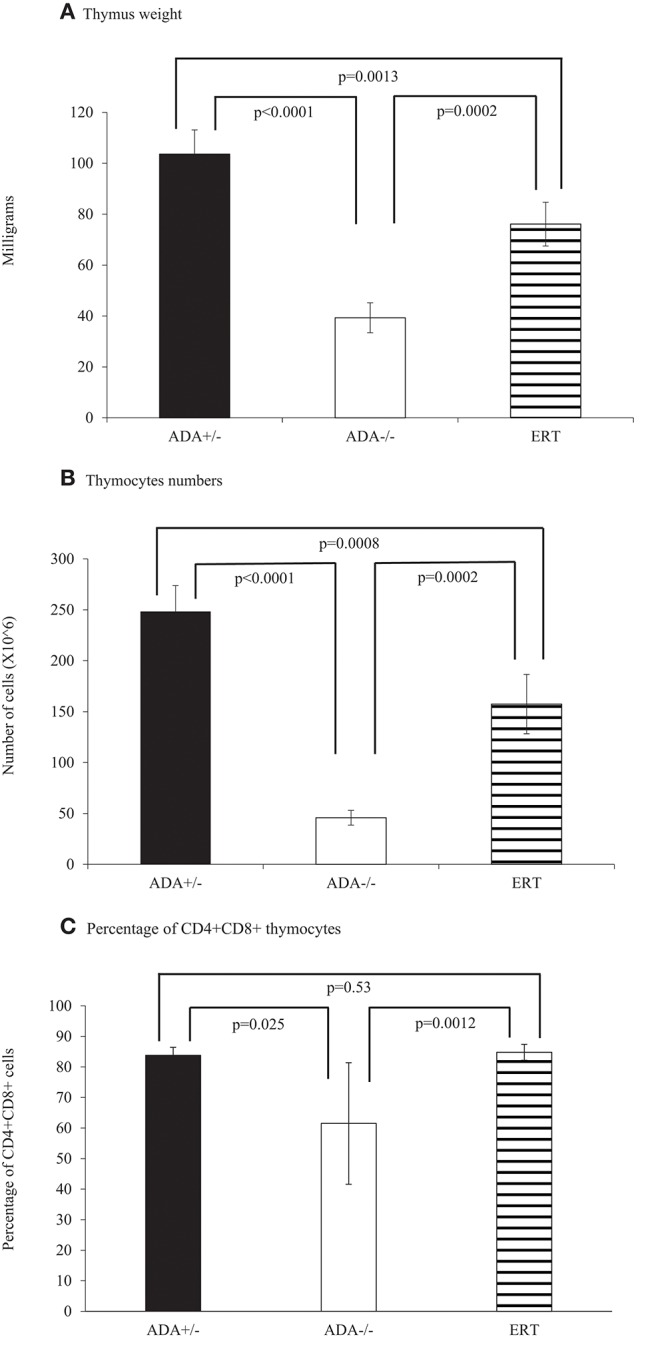
Thymus analysis of 17 days pp ADA+/– mice, ADA–/– mice, and ADA–/– mice following ERT. Thymus weight **(A)**, thymocytes numbers **(B)**, and percentages of CD4+CD8+ thymocytes **(C)** of 17 days postpartum (pp) ADA+/– healthy littermate control mice (filled squares), ADA–/– mice (open squares), and ADA–/– mice that received ERT initiated at 7 days pp (stripe pattern). Results are the mean and standard deviation of 3 independent experiments of 4–6 mice in each group.

**Figure 5:**
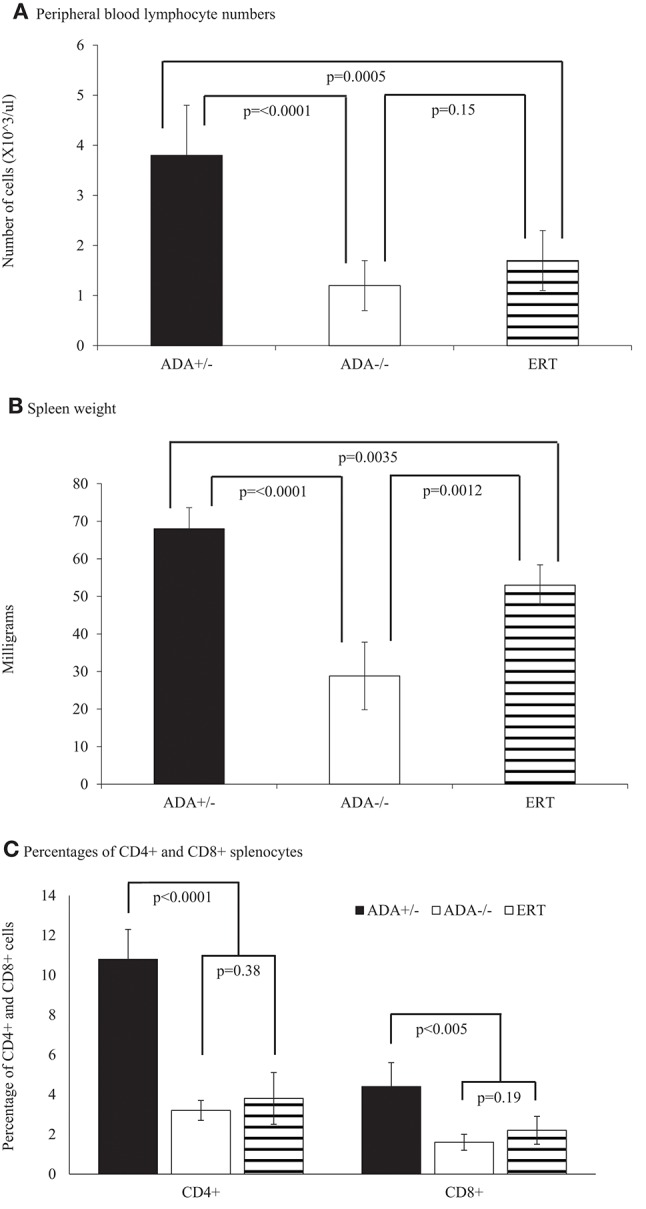
Lymphocytes and spleen analysis of 17 days pp ADA+/– mice, ADA–/– mice, and ADA–/– mice following ERT. Lymphocyte numbers **(A)**, spleen weight **(B)** and percentages of CD4+ and CD8+ splenocytes **(C)** of 17 days postpartum (pp) ADA+/– healthy littermate control mice (filled square), ADA–/– mice (open square), and ADA–/– mice that received ERT initiated at 7 days pp (stripe pattern). Results are the mean and standard deviation of 3 independent experiments of 4–7 mice in each group.

The ability of ADA-proficient maternal placenta and maternal blood cells' to uptake and detoxify Ado and dAdo is thought to protect ADA-deficient fetuses from the effects of abnormal purine metabolism. In ADA-deficient patients, Ado and dAdo accumulate quickly after birth, causing a rapid decline in T cells and lymphocytes in the first days of life (20). To determine whether a similar effect occurs in ADA–/– mice, we first assessed the thymus and spleen of ADA–/– mice shortly after birth. A significant reduction (*p* = 0.0073) in the number of thymocytes in ADA–/– mice (7.9 ± 2.7 × 10^∧^6 cells) compared to littermates (24.1 ± 8.4 × 10^∧^6 cells) was already evident at 3–4 days pp. Similarly, the percentages of CD4+CD8+ thymocytes, 57.0 ± 13.7% in ADA–/– mice were significantly reduced compared to the 79.7 ± 2.1% in ADA+/– mice. However, there was no difference in the weights of the thymus or spleen between ADA–/– and ADA+/– mice, nor were there differences in the numbers of CD4+ or CD8+ T cells in the spleen (data not shown). By 7 days pp, the weights of the thymus in ADA-/- mice (24.6 ± 10.3 mg) were significantly reduced (*p* = 0.0051) compared to healthy littermates (48.5 ± 12.3 mg). The decline in thymus weights (Figure 4A), thymocytes numbers (Figure 4B), and CD4+DC8+ thymocytes (Figure 4C), progressed and became more pronounced in ADA–/– mice by 17 days pp (*p* < 0.0001). Moreover, at 17 days pp the number of lymphocytes in the peripheral blood (Figure 5A), as well as the weight of the spleen (Figure 5B) and the numbers of CD4+ and CD8+ T cells in the spleen (Figure 5C), were significantly decreased in ADA–/– mice compared to ADA+/– littermates.

**Figure 6:**
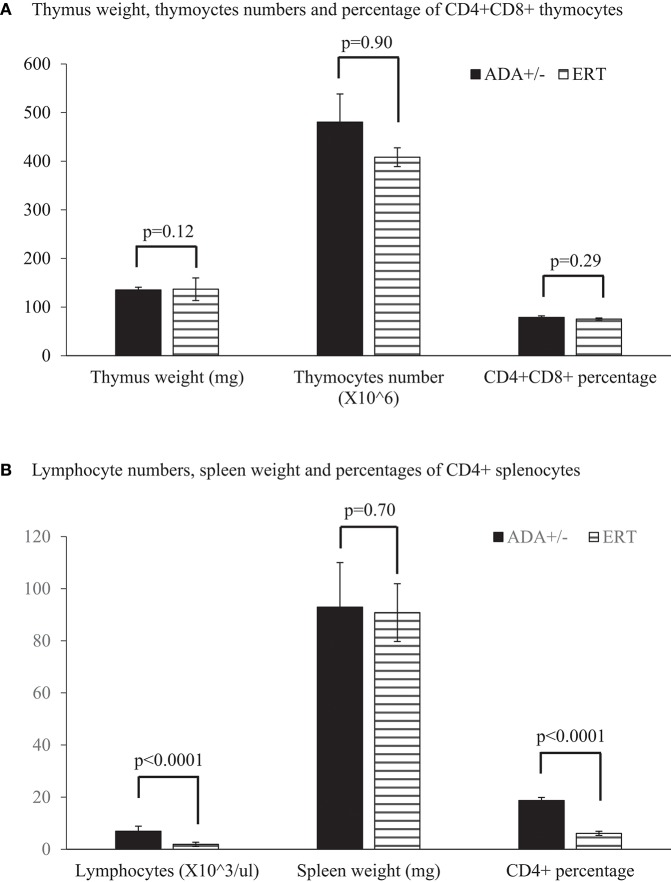
Thymus, lymphocytes, and spleen analysis of 35 days pp ADA+/– mice and ADA–/– mice following ERT. Thymus weight, thymoyctes numbers, and percentage of CD4+CD8+ thymocytes **(A)**, as well as lymphocyte numbers in peripheral blood, spleen weight, and percentages of CD4+ splenocytes **(B)** of 35 days postpartum (pp) ADA+/– healthy littermate control mice (filled square) and ADA–/– mice that received ERT initiated at 7 days pp (stripe pattern). Results are the mean and standard deviation of 3 independent experiments of 4–7 mice in each group.

Early identification of ADA deficiency through NBS allows rapid initiation of ERT, often within the patients' first weeks of life. To simulate this scenario, and study the benefits of early ADA supplementation, ADA–/– mice received PEG-ADA twice weekly from 7 days pp, till their assessment. At 17 days pp, ERT treated ADA-/- mice had significant increases in thymus weight (Figure 4A), numbers of thymocytes (Figure 4B) and percentages of CD4+CD8+ thymocytes (Figure 4C) in comparison to untreated ADA–/– mice, although not to normal levels. In contrast, the effects of ERT on peripheral blood lymphocytes (Figure 5A), the spleen weights (Figure 5B) as well as the number of CD4+ and CD8+ T cells (Figure 5C) in the spleen of ADA–/– mice were only partial, as these values remained significantly lower than normal littermates. Continuation of ERT until 35 days pp resulted in nearly normal thymus weight, number of thymocytes and percentage of CD4+CD8+ thymocytes (Figure 6A). In contrast, the number of lymphocytes in the blood and CD4+ cells in the spleen of 35 days pp ADA–/– mice who received ERT remained significantly reduced compared to ADA+/– littermates control mice, although spleen weight had normalized (Figure 6B).

## Discussion

ADA deficiency, recognized already in 1972 (31), is among the most common causes of SCID, yet the effects of this systemic metabolic disease are still being unraveled (14, 32). Hearing abnormalities have been reported in ADA-deficient patients, however, the lack of prospective studies on large cohorts of patients and presence of confounding factors such as infections, use of ototoxic medications, and coexistence of neuro-developmental defects have hindered better understanding of such association. To better answer this clinically relevant question, we took advantage of the well-established ADA–/– mice model, shown previously to recapitulate many of the features of ADA-deficient patients (21). Here we demonstrate for the first time that ADA–/– mice also suffer from severe hearing loss from a very young age. We also show that ADA deficiency is a direct cause of the auditory abnormalities as ADA–/– mice developed hearing loss shortly after birth, even when kept in a pathogen-free environment and without exposure to medications or evidence of infections. Hence, we add another non-infectious complication caused by ADA deficiency to the growing list of systemic effects of abnormal Ado metabolism.

The identification of the hearing abnormalities in ADA–/– mice also provided us with a tool to further investigate potential mechanisms for this phenomenon. ADA-deficient patients frequently suffer from lung disease while ADA–/– mice develop rapid respiratory failure (22). Also, we had previously shown that inner hair cells are susceptible to hypoxia (33, 34). Therefore, we evaluated the possible contribution of progressive systemic hypoxia to the hearing defect in ADA–/– mice. While oxygen treatment normalized oxygen saturation in the blood of ADA–/– mice, the hearing abnormalities persisted, suggesting that the systemic hypoxia is an unlikely explanation for the defect. Nevertheless, it is still possible that localized vascular abnormalities in the cochlea, such as the vasoconstriction seen in kidneys exposed to Ado (35), contribute to local hypoxia. Developmental delay and brain abnormalities have frequently been reported in ADA-deficient patients (11). To investigate whether delayed maturation of hearing pathways was responsible for the auditory dysfunction, we treated ADA–/– mice with ERT until 21 days pp, followed by re-exposure of the mice to altered Ado environment by withholding ERT. Remarkably, within less than 2 weeks, the ADA–/– mice exhibited severe hearing loss, demonstrating that impaired development was not the culprit.

SEM revealed marked abnormalities in the cochlear hair cells and stereocilia of ADA–/– mice. The development of the cochlea has been shown previously to depend on tight control of the concentration of Ado and its phosphorylated metabolite, ATP (36). Elevated concentrations of Ado have been found in the brain of ADA–/– mice (11, 23), and it is possible that the damage is related to elevated dATP, although a previous study in 12 ADA-SCID patients showed no apparent correlation between dATP at the time of diagnosis and deafness (12). Additionally, the preferential destruction of the hair cells in the apex of the cochlea and the low-frequency hearing defects in ADA–/– mice are reminiscent of the findings in mice with Wolframin deficiency, caused by mutations in the WFS1 gene (37). Wolframin is thought to control protein folding and maintenance of endoplasmic reticulum function by regulating calcium levels. Hence, abnormalities in calcium, due to impaired signaling through G-protein-coupled Ado receptors expressed on cochlear cells, might also be responsible for the sensory neural hearing abnormalities in ADA deficiency. Notably, cochlear hair cells were analyzed at 17 days pp, while it is possible that damage to the cochlea occurred already at an earlier age, similar to the effects of ADA deficiency on the lymphoid lineage. Future studies, possibly with hair cell-like cells expanded from patient's induced pluripotent stem cells, as recently utilized by others (38), might provide a better appreciation of the isolated effects of ADA deficiency on otic cells.

Importantly, early ADA ERT ameliorated the development of hearing abnormalities and cochlear defects in ADA–/– mice, concurrent with the benefits observed in the lungs and other tissues of treated mice. Yet, whether similar benefits can also be achieved in ADA-deficient patients remains to be determined. The early regimen also entailed administration of ERT twice a week, compared to weekly administration with the “late” regimen, hence the higher cumulative PEG-ADA dosing and better detoxification could have also had a role in the cochlear protection in the mice. ERT in ADA–/– mice began prior to hearing onset, while among ADA-deficient patients, several weeks of exposure to impaired purine metabolites before ERT is established might result in irreversible ototoxicity. Nevertheless, the encouraging results presented here should prompt early intervention for the prevention of hearing abnormalities in ADA-SCID.

Further support for the need to start ERT as early as possible was also evident by the independent effects on the T lineage. After confirming that similar to ADA-deficient patients, significant thymus abnormalities are evident by 3–4 days pp in ADA–/– mice, we showed that early ERT led to marked improvement in thymus development, reflected by increased weight and thymocyte numbers, as well as thymocytes maturation, which was evident by increased percentages of double positive cells. This important benefit is likely due to the reversal of the abundant T cell apoptosis previously identified in the thymus of ADA–/– mice (39). However, the number of lymphocytes in the blood, the spleen weight and T cells in the spleen did not increase significantly with ERT. The results of our study expand those of a previous report of the marked improvement in thymocytes numbers and subpopulations, but not in splenocytes or T and B cells in spleen in 16 days old ADA–/– mice treated with PEG-ADA (25). Interestingly, while continuing ERT for 5 weeks in ADA–/– mice normalized thymus weight, thymocyte numbers, and CD4+CD8+ cells, as previously shown (24), our study adds the finding that the number of lymphocytes in the blood and the percentages of CD4+ T in the spleen remain markedly reduced. Whether higher doses or longer duration of ERT will also reverse these abnormalities remains to be determined. Nevertheless, our study emphasizes again the important benefit of early ADA ERT for the T lineage development in ADA deficiency.

In conclusion, we demonstrate here that ADA–/– mice develop hearing deficits and cochlear damage as well as lymphoid abnormalities shortly after birth that can be corrected by early ERT. Further studies are needed to determine whether such benefits can also be achieved by rapid initiation of ERT in ADA-SCID patients.

## Data Availability

All datasets generated for this study are included in the manuscript and/or the supplementary files.

## Author Contributions

XX, JN, WM, and MT performed the experiments. MP, RH, and EG conceived and designed the experiments, analyzed the results, and oversaw the project. All authors discussed the experiments. JN, WM, and RH conducted and analyzed the ABR and SEM. XX and MT conducted and analyzed the immunological studies. EG wrote the main text of the manuscript and all authors reviewed and contributed to the preliminary and final draft of the manuscript.

### Conflict of Interest Statement

The authors declare that the research was conducted in the absence of any commercial or financial relationships that could be construed as a potential conflict of interest.

## Abbreviations

ABR: Auditory brainstem evoked responses
ADA: Adenosine deaminase
ADA-SCID: Adenosine deaminase induced severe combined immunodeficiency
Ado: Adenosine
dAdo: deoxyadenosine
ERT: Enzyme replacement therapy
HSCT: Hematopoietic stem cell transplantations
NBS: Newborn screening
PEG-ADA: Polyethylene glycol conjugated adenosine deaminase
PP: Post-partum
SCID: Severe combined immunodeficiency
SEM: Scanning electron microscopy.
